# Social network methods for HIV case‐finding among people who inject drugs in Tajikistan

**DOI:** 10.1002/jia2.25139

**Published:** 2018-07-22

**Authors:** Maxim Kan, Danielle B Garfinkel, Olga Samoylova, Robert P Gray, Kristen M Little

**Affiliations:** ^1^ Regional Monitoring & Evaluation Advisor, Population Services International (PSI)/Central Asia Almaty Kazakhstan; ^2^ Program Analytics PSI Washington DC USA; ^3^ PSI/Central Asia Almaty Kazakhstan; ^4^ PSI Washington DC USA; ^5^ HIV/TB Department PSI Washington DC USA

**Keywords:** HIV, AIDS, Central Asia, Tajikistan, case‐finding, network, respondent driven sampling

## Abstract

**Introduction:**

HIV testing programmes have struggled to reach the most marginalized populations at risk for HIV. Social network methods such as respondent‐driven sampling (RDS) and peer‐based active case‐finding (ACF) may be effective in overcoming barriers to reaching these populations. We compared the client characteristics, proportion testing HIV positive (yield), and number of new cases found through two RDS strategies and an ACF approach to HIV case‐finding among people who inject drugs (PWID) in Tajikistan.

**Methods:**

Routine programme data from adult PWID recruited to testing under the HIV Flagship Project in Tajikistan were analysed to compare client demographic and clinical characteristics across the three approaches. We also compared the number of previously untested clients, the number of new HIV cases found, and the yield across the case‐finding strategies, and evaluated predictors of new HIV diagnosis using fixed‐effects logistic regression.

**Results:**

From 24 October 2016 to 30 June 2017, Flagship tested 10,300 PWID for HIV, including 2143 under RDS with unrestricted waves (RDS1, yield: 1.5%), 3517 under restricted RDS (RDS2, yield: 2.6%), and 4640 under ACF (yield: 1.5%). Clients recruited under ACF were similar in age (35.8 vs. 36.8) and gender (91% vs. 90% male) to those recruited through RDS, though ACF clients were more likely to report being a first‐time tester (85.1% vs. 68.3%, *p* < 0.001). After controlling for age, sex, previous testing history and accounting for clustering at the site level, we found that clients tested under both RDS1 (aOR: 1.74, 95% CI: 1.04 to 2.90) and RDS2 (aOR: 1.54, 95% CI: 1.11 to 2.15) had higher odds of testing newly positive for HIV relative to clients recruited through ACF. We did not find significant differences in the odds of new HIV infection between those recruited from RDS1 versus RDS2 (aOR: 1.12, 95% CI: 0.67 to 1.86).

**Conclusions:**

RDS‐based interventions resulted in higher yields and overall case‐finding, especially when recruitment was restricted. However, ACF identified a higher proportion of first‐time testers. To find at least 90% of PWID living with HIV in Tajikistan, it may be necessary to implement multiple case‐finding approaches concurrently to maximize testing coverage.

## 1 Introduction

While national HIV prevalence in Tajikistan is 0.3%, prevalence among the estimated 23,000 people who inject drugs (PWID) in the country is 13.5% 1. Though PWID represent <0.3% of the total population in Tajikistan, they make up approximately 19% of all PLHIV in the country 2. Data also suggest that HIV testing coverage among PWID remains inadequate 3 to meet the UNAIDS 90‐90‐90 goals. There is an urgent need to expand testing services and treatment among PWID in Tajikistan, who are disproportionately impacted by HIV.

To improve HIV case‐finding among PWID in Tajikistan, the USAID Central Asia HIV Flagship Project (Flagship) aims to rapidly scale‐up HIV case‐finding and linkage to care using two methods: respondent driven sampling (RDS) and active case‐finding (ACF). RDS was originally designed as a technique for generating a representative sample in hidden or hard‐to‐reach populations for which a sampling frame is not available 4. RDS uses a snowball‐sampling approach 5 in which initial “seeds” are recruited and provided with coupons to distribute to their peers.  Seeds typically receive an incentive for each peer recruited, who are in turn provided with a small, primary incentive, and given additional coupons to recruit their peers. When these coupons are returned, a small secondary incentive is provided to the recruiter.  Recruitment continues as such until the required sample size is reached 6.

As a recruitment technique, RDS is susceptible to bias 7, in part because recruiters tend to recruit others like themselves 7, 8. Researchers have hypothesized that these biases could be leveraged to over‐recruit from high‐risk networks, thereby using RDS as an effective HIV case‐finding technique 9, 10. Because “like recruits like,” RDS may be used to find new cases of HIV by having PLHIV recruit others from their social network, who themselves are more likely to be PLHIV. Previous research with PWID in Mexico tested this idea and found HIV‐infected PWID were more likely to recruit other PLHIV than their HIV‐uninfected peers 9.

While the potential of RDS for HIV case‐finding has been explored 11, less is understood about the optimal approach to implementation under programmatic conditions, including the number of waves of recruitment that should be undertaken and the number of coupons that should be distributed to HIV‐negative and positive recruits. To better understand the optimal strategy at scale, Flagship deployed two RDS strategies, as well as a peer‐based ACF intervention, during the first nine months of programme implementation. Under the first approach (RDS1), recruitment could continue indefinitely, with each PWID tested receiving additional coupons to recruit members of his/her social network. Under the second approach (RDS2), recruitment was stopped after two HIV‐negative waves (e.g. if two successive individuals were recruited who were HIV‐negative, no coupons were provided for further recruitment). Under the ACF intervention, “Peer Navigators” (PN) – individuals who were themselves living with HIV, on treatment, or who were current/former PWID – recruited their peers for HIV testing services (HTS) through direct outreach. These same PNs also recruited seeds for the RDS interventions and provided case‐management services to PLHIV identified by the project (Additional file 1).

Using routine programmatic data from the Flagship Project's monitoring information system (MIS), we evaluated the yield, number of new HIV cases found, and the demographic characteristics (age, sex, HIV testing history, etc.) of those tested under each approach. The findings are intended to inform the scale‐up of social network interventions for HIV case‐finding among PWID in Tajikistan, and the implementation of other social‐network based interventions targeting hard‐to‐reach populations.

## 2 Methods

### 2.1 Programme population

We analysed routine programmatic Flagship data from three sub‐national units (Dushanbe City, Districts of Republican Subordination (DRS), and Sughd Oblast) in Tajikistan from 24 October 2016 to 30 June 2017. Subjects included seeds and recruits identified through RDS1, RDS2, and ACF. Eligibility criteria under the project included age ≥18, self‐report of injecting drugs in the preceding six months and/or evidence of injection drug use (e.g. track‐marks), and no self‐reported HIV testing in the preceding six months.

All clients recruited to the programme were screened for eligibility and basic demographic data (e.g. age, sex, previous HIV testing and results) were collected using a paper‐based programme intake form from which de‐identified data were entered into the project MIS. Clients were tracked using a unique identifier code (UIC), and no personal client identifiers (e.g. name, phone number, address) were included in the database. Data were collected as a part of routine service delivery, and the Population Services International Research Ethics Board granted a non‐research determination for this analysis. Flagship clients provided verbal consent for HIV testing and the collection of health‐related data.

### 2.2 Project design and procedures

Under both RDS1 and RDS2, PNs were instructed to select seeds with large social networks who would be committed to coupon distribution. Seeds were primarily PWID living with HIV and were selected to reflect underlying population diversity in terms of residence, age, sex, and duration of injecting behaviour. Seeds were provided with three coupons to distribute to PWID in their networks. Seeds were instructed to recruit those who had not had a recent HIV test (defined as not having tested within the preceding six months).

Recruits were escorted for HTS at AIDS Centers or underwent rapid‐testing in the community. Recruits received a small non‐cash incentive (equivalent to $3 USD) after testing, as well as three recruitment coupons, and instructions on who to recruit and how to do so. Recruiters received an additional non‐cash incentive (equivalent to $1.50 USD) for each additional member of their networks who redeemed a coupon and underwent testing. RDS1 recruitment could continue indefinitely, and no limits were placed on the number of successive waves. RDS2 recruitment ceased after two HIV‐negative waves but could continue in chains where a recruit tested HIV‐positive.

The ACF approach was based on the concept of continuously sourcing new networks of PWID and offering them HIV testing. PWID working as ACF PNs offered HTS to other PWID through community outreach, prioritizing communities with large numbers of PWID and low HIV testing coverage. PNs were given weekly targets for new PWID tested and new HIV cases found, with targets managed closely through supportive supervision. Clients recruited through ACF also received a small, non‐cash incentive (equivalent to $3 USD) after testing.

Any PWID testing positive were linked to confirmatory HTS at the government AIDS Center. Those confirmed HIV‐positive were enrolled into Flagship's case management programme and linked to public sector care and treatment. Clients testing HIV‐negative were provided with referrals to prevention services, including opioid substitution therapy (OST) and needle and syringe programmes (NSPs).

### 2.3 HIV testing

HTS occurred through two channels: referrals to AIDS centres (from October 2016 to June 2017) and rapid testing at non‐governmental organizations (NGOs) or in the community (since April 2017). Prior to introducing rapid testing, clients were referred to AIDS centres for testing conducted according to the national algorithm 12. After rapid tests were introduced, trained PNs tested clients using OraQuick HIV ½ Rapid Antibody test kits, (OraSure Technologies, Inc., Bethlehem, PA, USA). Rapid tests were performed at implementing NGOs under RDS and by trained PNs in the field for ACF. All clients testing positive on a rapid test were escorted to an AIDS centre for confirmatory testing according to the national algorithm 12. Clients diagnosed with HIV were traced in the national treatment database by AIDS centre staff at the time of confirmatory testing to identify clients already on pre‐antiretroviral therapy (ART)/ART. Clients not already registered in the national database were considered “newly diagnosed.” Identifiable data used for tracing were not collected by the Flagship project, and were not entered into the project MIS.

### 2.4 Statistical analysis

We compared the number of participants tested, the number/proportion of new testers, the number/proportion of women tested, the age of recruits, the number of new HIV cases found, and the yield across the three approaches using Pearson's χ^2^ test or Fisher's exact test (for binary variables) and t‐tests or Wilcoxon rank sum tests (for continuous variables). Yield was defined as the proportion of clients who were HIV‐positive on their first test, divided by the total number of respondents tested (excluding seeds). We also evaluated the characteristics of recruitment effectiveness by comparing HIV‐positive and HIV‐negative recruits. To ensure comparability across approaches, we analysed all programme data from 4 to 5 months of implementation for each of the three approaches.

Predictors of new HIV diagnosis were evaluated using fixed‐effects logistic regression 13, with a term to account for clustering at site level. A univariate model comparing odds of new HIV infection across the three recruitment strategies was fitted, and demographic factors were added based on univariate significance of <0.10 and/or programmatic or epidemiological importance. Model fit was compared using Bayesian information criterion, and variance inflation factors were evaluated to check for multicollinearity. All analyses were performed using STATA 13.0 (Stata Corporation, College Station, TX, USA).

## 3 Results

### 3.1 Description of recruits across methods

Flagship tested a total of 10,300 PWID for HIV in Tajikistan between 24 October 2016 and 30 June 2017 (Table 1). Altogether 181 seeds and 2143 (20.8%) PWID recruits were tested under RDS1, compared to 264 seeds and 3517 (34.1%) recruits under RDS2. A total of 4640 (45.0%) PWID were tested under the ACF approach. Flagship diagnosed 190 preliminary positive HIV cases, for an overall yield of 1.8%. Of these, Flagship PNs escorted 152 (80%) new preliminary positive HIV cases to confirmatory testing, and 133 of these (87.5%) were initiated onto treatment. Most PWID were recruited from Sughd Oblast (4399, 42.7%), with another 3211 (31.2%) from the DRS, and 2690 (26.1%) from Dushanbe City. Most HIV cases were from Sughd Oblast (117/190, 61.6%), with a smaller proportion from the DRS (42, 22.1%) and Dushanbe City (31, 16.3%). Overall yield ranged from 1.2% in Dushanbe City to 2.7% in Sughd Oblast.

**Table 1:** Demographic and clinical characteristics of people who inject drugs recruited to HIV testing in Tajikistan

Variable	Total (N = 10,300)	RDS1 (n = 2143, 20.8%)	RDS2 (n = 3517, 34.2%)	ACF (n = 4640, 45.1%)	*p*‐value
	N (%)	n (%)	n (%)	n (%)	
Female	1001 (10.4)	279 (13.0)	311 (8.8)	411 (8.9)	<0.001
Geography					<0.001
Dushanbe City	2690 (26.1)	739 (34.5)	1011 (28.8)	940 (20.3)	
Districts of Republican Subordination	3211 (31.2)	434 (20.3)	681 (19.4)	2096 (45.2)	
Sughd Oblast	4399 (42.7)	970 (45.3)	1825 (51.9)	1604 (34.6)	
Age (mean, standard deviation (SD))	36.4 (8.8)	37.9 (9.2)	36.1 (8.5)	35.8 (8.7)	<0.001
Non‐government organization					<0.001
Bovari	552 (5.4)	552 (25.8)	0 (0.0)	0 (0.0)	
Marvorid	937 (9.1)	336 (16.2)	188 (5.4)	403 (8.7)	
Nasli Javoni	651 (6.3)	651 (30.4)	0 (0.0)	0 (0.0)	
Spin Plus	2138 (20.8)	187 (8.7)	1011 (28.8)	940 (20.3)	
Dina	2328 (22.6)	209 (9.8)	1016 (28.9)	1103 (23.8)	
Buzurg	534 (5.2)	22 (1.0)	329 (9.4)	183 (3.9)	
Guli Surkh	1631 (15.8)	45 (2.1)	249 (7.1)	1337 (28.8)	
Rokhi Zindagi	886 (8.6)	88 (4.1)	480 (13.7)	318 (6.9)	
Tajikistan Network	643 (6.2)	43 (2.0)	2440 (6.9)	356 (7.7)	
Recruitment wave					<0.001
1	1275 (22.5)	406 (19.0)	869 (24.7)	–	
2	3264 (57.3)	823 (38.4)	2441 (69.4)	–	
3	599 (10.6)	410 (19.1)	189 (5.4)	–	
4	305 (5.4)	287 (13.4)	18 (0.5)	–	
5	137 (2.4)	137 (6.4)	0 (0.0)	–	
6	58 (1.0)	58 (2.7)	0 (0.0)	–	
7	13 (0.2)	13 (0.6)	0 (0.0)	–	
8	5 (0.1)	5 (0.2)	0 (0.0)	–	
9	4 (0.1)	4 (0.2)	0 (0.0)	–	
Never tested for HIV	7818 (75.9)	1448 (67.6)	2420 (68.8)	3950 (85.1)	<0.001
Shared needles with recruiter	1398 (24.7)	620 (28.9)	778 (22.1)	–	<0.001
Had sex with recruiter	123 (2.2)	58 (2.7)	65 (1.9)	–	0.032
Migration experience	5374 (52.2)	923 (43.0)	1743 (49.6)	2706 (58.3)	<0.001
Network size (mean, SD)	7.7 (6.4)	7.5 (6.4)	7.8 (6.5)	–	0.034
HIV positive	190 (1.8)	32 (1.5)	90 (2.6)	68 (1.5)	0.001

RDS1, respondent‐driven sampling with unrestricted recruitment; RDS2, RDS with restricted recruitment (in which recruitment is stopped after two HIV‐negative waves, but allowed to proceed otherwise); ACF, active case‐finding (peer‐led, community‐based HIV case‐finding).

**Figure 1:**
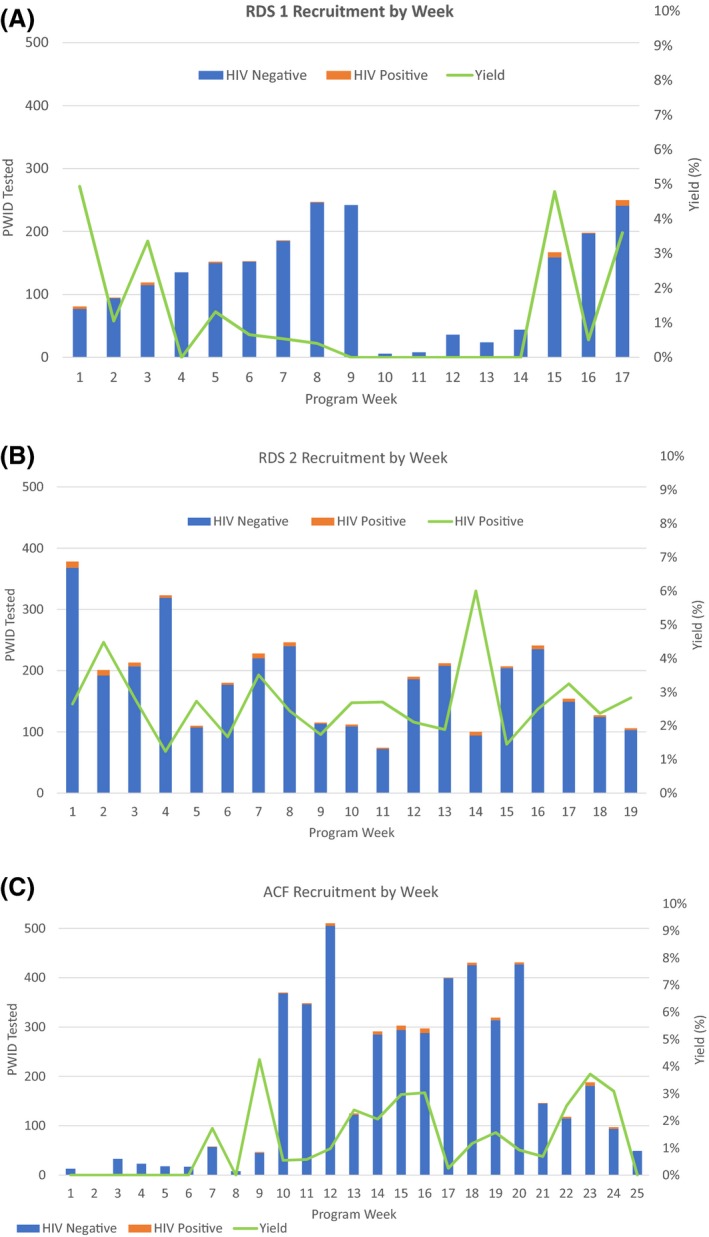
**(A‐C)** Recruitment across three approaches to HIV case‐finding amongst people who inject drugs in Tajikistan. RDS, respondent driven sampling; RDS1, RDS with unrestricted recruitment; RDS2, RDS with restricted recruitment (in which recruitment is stopped after two HIV‐negative waves, but allowed to proceed otherwise); ACF, active case‐finding (peer‐led, community‐based HIV case‐finding approach). Figures show the number of clients recruited to testing each week of intervention implementation. The weekly yield of case‐finding activities (the number of clients testing positive for HIV divided by the number of clients tested) is shown in green.

RDS1 was conducted between 24 October 2016 to 21 February 2017, recruiting an average of 17.9 clients daily (Figure 1A). Altogether 32 (1.5%) RDS1 clients were newly diagnosed with HIV. RDS2 ran from 22 February 2017 to 30 June 2017, with an average daily recruitment rate of 27.5 clients (Figure 1B). A total of 90 (2.6%) RDS2 clients were newly diagnosed with HIV. ACF ran from 9 January 2017 to 30 June 2017 and tested an average of 27 clients per day. The ACF approach identified 68 (1.5%) newly diagnosed HIV cases (Figure 1C). Yield was similar between RDS1 and ACF (1.54% vs. 1.47%, *p* = 0.414), but was slightly higher under RDS2 compared to ACF (2.6% vs. 1.5%, *p* < 0.001).

Most Flagship clients were male (87.6%) (Table 1), though the proportion of females was higher among those tested under RDS1 (13.0%) compared to ACF (8.9%) and RDS2 (8.8%) (*p* < 0.001). Clients averaged 36.3 years of age. Clients recruited through ACF were slightly younger on average (35.8 years) than those recruited through RDS (RDS1: 37.9; RDS2: 36.1, *p* < 0.001). Approximately, 68% of clients reached through RDS were self‐reported first‐time testers, compared to 85% of ACF clients (*p* < 0.001).

### 3.2 Yield across approaches

Yield among females was higher than among males for both RDS (4.6% for females vs. 1.9% for males, *p* < 0.001) and ACF (4.4% vs. 1.2%, *p* < 0.001) (Table 2). Among self‐reported new testers, testing yield was higher under RDS than ACF (2.4% vs. 1.4%, *p* = 0.002), though no differences were observed in yields between ACF and RDS among those who had previously tested (1.7% vs. 1.7%, *p* = 0.910).

**Table 2:** Comparison of HIV testing yield^a^ between respondent‐driven sampling and active case‐finding among people who inject drugs in Tajikistan

Variable	RDS^b^ % (n) N = 5660	ACF^c^ % (n) N = 4640	*p*‐value
Total	2.2 (122)	1.5 (68)	0.009
Male	1.9 (95)	1.2 (50)	0.007
Female	4.6 (27)	4.4 (18)	0.883
Age 18 to 30 years	1. 3 (19)	1.3 (17)	0.962
Age >30 years	1.5 (51)	2.4 (103)	0.004
District of Republican Subordination	1.9 (21)	1.0 (21)	0.036
Dushanbe City	1.3 (23)	0.9 (8)	0.283
Sughd Oblast	2.8 (78)	2.4 (39)	0.476
Never tested for HIV	2.4 (92)	1.4 (56)	0.002
History of migration	2.1 (57)	1.4 (38)	0.042

a Yield was defined as the proportion of clients testing positive on their first HIV test out of all clients tested for HIV;

b RDS includes the aggregate results from respondent driven sampling approach 1 (RDS1) with unrestricted waves, and RDS2 (in which recruitment was stopped after 2 HIV‐negative waves);

c ACF, active case‐finding approach (utilized peer‐outreach workers to perform community‐based HIV case‐finding).

Yield was higher among RDS clients who reported having had sex with their recruiter (6.5% vs. 2.0%, *p* = 0.001) or sharing a needle with their recruiter (3.7% vs. 1.5%, *p* = 0.009). Yield was higher among clients testing under RDS2 versus RDS1 across most demographic groups (Table 3), including men and previous testers. HIV‐positive recruiters had a higher overall yield among those they directly recruited than HIV‐negative recruiters (5.3% vs. 2.6%, *p* = 0.002).

**Table 3:** Comparison of HIV testing yield^a^ between respondent‐driven sampling with restricted and unrestricted recruitment waves among people who inject drugs in Tajikistan

Variable	RDS1 N = 2143 % (n)	RDS2 N = 3517 % (n)	*p*‐value
Total	1.5 (32)	2.6 (90)	0.007
Male	1.3 (24)	2.2 (71)	0.019
Female	2.9 (8)	6.1 (19)	0.060
Sughd Oblast	1.4 (14)	3.5 (64)	0.002
Dushanbe City	8 (110)	1.5 (15)	0.467
District of Republican Subordination	2.3 (10)	1.6 (11)	0.409
Ever tested for HIV	0.9 (6)	2.2 (24)	0.033
Never tested for HIV	1.8 (26)	2.7 (66)	0.066
Shared needle with recruiter	1.5 (9)	3.7 (29)	0.009
Had sex with recruiter	0.0 (0)	12.3 (8)	0.006
Never had sex with recruiter	1.5 (32)	2.4 (81)	0.038
No history of migration	1.2 (15)	2.8 (50)	0.003

RDS1, respondent‐driven sampling with unrestricted recruitment; RDS2, RDS with restricted recruitment (in which recruitment is stopped after two HIV‐negative waves, but allowed to proceed otherwise).

a Yield was defined as the proportion of clients testing positive on their first HIV test out of all clients tested for HIV.

### 3.3 Seed characteristics

Altogether 445 seeds were recruited for RDS, including 181 (40.7%) under RDS1 and 264 (59.3%) under RDS2 (Table 4). Seeds were majority HIV positive (94.8%), male (84.0%), and averaged 40.2 years old. Most seeds were married (51%) and recruited through community networks (64.9%) or referred by the AIDS Centre (32.7%). Altogether 200 (44.9%) seeds were launched in Sughd Oblast, 95 (21.4%) in DRS, and 150 (33.7%) in Dushanbe City. PLHIV seeds had smaller average self‐reported network sizes compared to HIV‐negative seeds (11.1 vs. 20.8, *p* < 0.001). Coupon redemption did not differ significantly between HIV‐positive seeds and HIV‐negative seeds (mean coupon redemption: 0.97 vs. 0.94, *p* = 0.579). Yield within HIV positive seed chains was 2.6% compared to 3.3% for chains started by HIV‐negative seeds (*p* = 0.571).

**Table 4:** Seed demographics, by HIV status and recruitment approach among people who inject drugs in Tajikistan

Variable	HIV‐negative seeds (n = 23, 5.2%)	HIV+ seeds (n = 422, 94.8%)	RDS1 (n = 181, 40.7%)	RDS2 (n = 264, 59.3%)	*p*‐value^a^
	n (%)	n (%)	n (%)	n (%)	
Female	1 (4.4)	70 (16.6)	29 (16.0)	42 (15.9)	0.010
Geography					0.060
Dushanbe City	0 (0.0)	150 (35.6)	61 (33.7)	89 (33.7)	
District of Republican Subordination	0 (0.0)	95 (22.5)	48 (26.5)	47 (17.8)	
Sughd Oblast	23 (100.0)	177 (41.9)	72 (39.8)	128 (48.5)	
Age (mean, standard deviation (SD))	38.1 (6.6)	40.4 (7.9)	40.8 (7.6)	39.9 (8.1)	0.240
Married	–	215 (51.0)	81 (44.8)	134 (55.6)	0.029
Migration experience	10 (43.5)	197 (46.7)	95 (52.5)	112 (42.4)	0.037
Network size (mean, SD)	11.1 (8.0)	20.8 (6.5)	9.4 (6.8)	18.7 (8.0)	<0.001
HIV positive	–	–	181 (100.0)	241 (91.3)	<0.001
Coupons redeemed (mean, SD)	0.94 (0.3)	0.97 (0.1)	0.94 (0.2)	0.95 (0.2)	0.579

RDS1, respondent‐driven sampling with unrestricted recruitment; RDS2, RDS with restricted recruitment (in which recruitment is stopped after two HIV‐negative waves, but allowed to proceed otherwise).

a *p*‐value compares the values between RDS1 and RDS.

### 3.4 Predictors of new HIV infection

On univariate analysis, recruitment through RDS1 or RDS2 (compared to ACF) was associated with increased odds of new HIV diagnosis (OR: 1.57, 95% CI: 1.15 to 2.16) (Table 5). Older age (OR: 1.03, 95% CI: 1.01 to 1.04) and female sex (OR: 3.37, 95% CI: 2.36 to 4.81) were also associated with increased odds of HIV infection. In the adjusted models, previous HIV testing (Model 2 aOR: 0.61, 95% CI: 0.42 to 0.90) and female sex (Model 2 aOR: 2.82, 95% CI: 1.72 to 4.64) were associated with odds of new HIV infection. Odds of a new HIV diagnosis did not differ significantly between those recruited through RDS1 versus RDS2 in any of the models.

**Table 5:** Fixed‐effects logistic regression model for HIV infection by recruitment strategy

Variable	Odds ratio (95% CI)	Adjusted odds ratio (95% CI)^a^
Model 1^a^		
Strategy		
RDS1	1.74 (1.04 to 2.90)	1.77 (1.05 to 2.98)
RDS2	1.54 (1.11 to 2.15)	1.67 (1.19 to 2.34)
Active case‐finding	Ref	Ref
Age	1.03 (1.01 to 1.04)	1.03 (1.01 to 1.05)
Female	3.37 (2.36 to 4.81)	3.58 (2.50 to 5.13)
Tested for HIV previously	0.72 (0.50 to 1.05)	0.61 (0.42 to 0.90)
Model 2^b^		
Strategy		
RDS1	1.12 (0.67 to 1.86)	1.12 (0.67 to 1.86)
RDS2	Ref	Ref
Age	1.02 (1.00 to 1.04)	1.03 (1.01 to 1.05)
Female	3.01 (1.91 to 4.76)	2.82 (1.72 to 4.64)
Tested for HIV previously	0.54 (0.34 to 0.85)	0.49 (0.31 to 0.79)
Had sex with recruiter	3.13 (1.44 to 6.80)	1.95 (0.85 to 4.46)
Shared needles with recruiter	1.26 (0.79 to 1.99)	1.25 (0.78 to 2.00)
History of migration	0.90 (0.61 to 1.35)	1.03 (0.68 to 1.56)

RDS1, respondent‐driven sampling with unrestricted recruitment; RDS2, RDS with restricted recruitment (in which recruitment is stopped after two HIV negative waves, but allowed to proceed otherwise).

a Model 1 adjusts for recruitment strategy (RDS1, RDS2, and ACF), age, sex, and HIV testing history and clustering at the non‐governmental organization (NGO) level.

b Model 2 adjusts for recruitment strategy (RDS1 and RDS2), age, sex, HIV testing history, history of sex with the recruiter, history of needle sharing with the recruiter and a history of migration, in addition to clustering at the NGO level.

## 4 Discussion

Strategies leveraging the social networks of PLHIV may improve the efficiency and effectiveness of HIV case‐finding programmes. Approaches such as index testing and assisted partner notification 14, as well as RDS methods 12 have shown high yields under programmatic conditions, and may be effective ways to close the testing gap, especially for hidden and hard‐to‐reach populations. Testing programmes also serve as important entry points for prevention services. Recent research suggests that interventions that link PWID to services such as OST and NSPs are likely to be very cost‐effective, if not cost‐saving, in Central Asian contexts, in addition to having a significant impact on HIV transmission and injecting behaviour 15.

The two variations on RDS‐based case‐finding presented here leveraged social networks in an effort to increase HIV case‐finding and linkages to treatment and prevention among PWID in Tajikistan. Together these approaches identified a total of 190 preliminary positive cases of HIV, linked 152 of these (80%) to confirmatory testing, and put 133 (87.5%) new clients on treatment. This represented a substantial proportion of all HIV tests conducted and new HIV cases found in the programme areas. Coupon generation, incentive delivery and tracking, and recruitment management were implemented by local NGOs, with recruitment and case‐management being performed by PNs who were themselves PLHIV or current/former PWID. Flagship experience demonstrates that RDS‐based methods are feasible approaches to HIV case‐finding among PWID in Tajikistan, and may be appropriate for other Central Asian countries and beyond. While yield and case‐finding varied across approaches, regular analysis of project data and timely adjustment of project strategies was crucial to maximize intervention impacts. Flagship is currently conducting analogous programmes in the Kyrgyz Republic, suggesting the scalability of this approach.

Similar HIV case‐finding projects have been conducted for key population testing programmes among MSM and PWID in India 16, high‐risk heterosexuals in the US 10 and PWID in Ukraine 17 and Vietnam 12. While some projects achieved testing yields of >10%, our yields ranged from 1.5% to 2.6%, well below the estimated PWID HIV prevalence in Tajikistan 1. It is unclear whether this was due to limitations in the case‐finding approach itself, declines in underlying prevalence of undiagnosed HIV among PWID (as the ART programme in Tajikistan has expanded), inaccurate HIV prevalence or size estimation data, or some other factor. Regardless, efforts to boost Flagship yields are underway, including differential provision of coupons to recruiters based on HIV risk characteristics (i.e. needle sharing, younger PWID, long‐term injectors, females, etc.), which may help boost enrolment of these sub‐populations. Further analysis of effective recruiters (e.g. those who recruited ≥1 HIV‐positive recruits) could inform differential targeting of coupon distribution. However, while yield rates were lower than expected, it should be noted that Flagship's case‐finding represented a substantial proportion of total new HIV cases diagnosed (and linked to treatment) in the programme areas, and these absolute numbers of new HIV cases found are vital to Tajikistan's reaching the ambitious 90‐90‐90 targets.

Our results suggest that recruits testing through RDS‐based case‐finding may differ in important ways from clients recruited through strategies like peer‐led ACF, including age, HIV testing history, and sex. While yield was lower under ACF compared to RDS2, ACF reached PWID clients more quickly than RDS1, did not require incentive payments for recruitment (though PWID received testing incentives), and reached a higher proportion of new testers. To work at maximum effectiveness, it may be necessary to implement multiple case‐finding approaches concurrently, such as assisted partner notification and RDS or ACF, or to conduct staggered RDS “campaigns” alongside other forms of case‐finding.

While approaches restricting recruitment of HIV‐negative recruits may increase yield (relative to unrestricted RDS), this increase in testing efficiency may require additional investments in seed recruitment, since recruitment chains are ended more quickly under this approach. At the time of analysis, RDS2 was beginning to exhaust its pool of PWID living with HIV available as new seeds, and there was some concern about continuing the pace of recruitment under this model. Further, after adjusting for site of recruitment and other demographic factors, we did not find a statistically significant difference in odds of new HIV infection between clients recruited through the two RDS approaches. However, we did find that RDS2 provided a more consistent flow of new clients over time, compared to the “boom and bust” of RDS1, allowing for more predictable allocation of staffing/resources. Regardless of the RDS method, additional seed recruitment requires considerable effort, and may slow down recruitment over time. Recruitment of high‐risk HIV‐negative seeds, especially for RDS2, may help to reduce this burden. The cost of seed recruitment between these approaches should be considered in future cost‐effectiveness research.

Strikingly, all three methods recruited insufficient numbers of female PWID, despite a high prevalence of undiagnosed HIV in this population. Female PWID, and female partners of male PWID, in Central Asia face multiple, intersecting HIV risks, and may be less able to negotiate consistent condom use, or to persuade a partner to go for couples HIV testing 18. Such women are also at high risk of gender‐based violence, and experience more HIV and drug‐related stigma and discrimination than their male counterparts 18. Given these risks, more efforts to reach female PWID with HTS, such as recruiting additional female seeds and PNs, are warranted. Similarly, efforts that successfully reach older and previously untested PWID appear likely to yield more HIV infections. Additional interventions, such as assisted partner notification 14, may further expand HIV case‐finding originating from network‐based methods. Finally, extension of these methods into other key populations, such as MSM, may help increase HIV testing coverage among other hard‐to‐reach populations with high HIV risks and low test coverage 19.

This analysis is subject to important limitations. Interventions were conducted concurrently in the same programme areas. Thus, differences across strategies may have been obscured, and outcomes may have varied if interventions had been implemented separately. While we did not find evidence of this, it is also possible that some respondents were tested multiple times in an effort to receive additional incentives. However, the World Health Organization (WHO) recommends at least annual testing for key populations, including PWID 20, and we judged the risk of any individual being tested multiple times within a six‐month period to be relatively low. The risk was minimized through use of a UIC. PNs, who were themselves members of the beneficiary populations, were also trained to spot clients coming for testing more than once semi‐annually. Such clients were provided HIV testing but did not receive an incentive.

Because RDS2 was conducted after RDS1, it is possible that the higher yield was driven partly by programmatic improvements independent of the RDS approach. While implementing partners were extensively trained and closely monitored, it is possible that actual implementation varied across organizations and time. We attempted to account for clustering at the organization level in our final model, but this analysis was limited by the small number of clusters. However, if these issues did have an effect, the authors feel the impacts likely bias findings towards the null. Because yield was calculated based on the first HIV test result (rather than confirmatory testing), yield may have been over‐estimated. Finally, this analysis was based on routine programmatic data, and some important covariates (e.g. years of injecting) were not collected.

Despite these limitations, this analysis contributes to a growing body of evidence about using RDS methods for HIV case‐finding among key populations. Other studies have explored strategies to improve the efficiency of these social network methods for HIV case‐finding 16, including the use of recency testing to identify newly HIV‐infected seeds 21 who may be indicative of risk networks in which HIV transmission is ongoing 17. While these studies have demonstrated the efficacy of network‐based approaches to case‐finding among key populations, ours is one of the few that has looked at the effectiveness of RDS‐based case‐finding at scale under programmatic conditions.

## 5 Conclusion

To reach the UNAIDS ambitious 90‐90‐90 targets by 2020, programmes worldwide need to find more effective ways of reaching those at highest risk of infection with testing. Flagship demonstrated the feasibility of conducting RDS for HIV case‐finding among PWID at scale, testing >5600 PWID in just eight months. While the yield from RDS‐based approaches was greater than that of the ACF approach, client profiles differed between the strategies, suggesting that multiple case‐finding approaches may be needed to ensure the first 90 target is met. Variations on RDS implementation, such as differential distribution of coupons, limiting recruitment after a number of HIV‐negative waves, or utilizing technologies like recency assays may increase testing yields, and should be considered by programme implementers. Decisions to scale‐up network‐based methods for HIV case‐finding depends on both the impact and cost‐effectiveness of these approaches. Future research should explore cost per case‐detected and cost‐effectiveness to better inform programmatic decision‐making. Future studies may also consider the ideal frequency of RDS‐based methods over time, comparing costs and health impact of an ongoing versus campaign‐style approach.

## Competing interests

The authors have no competing interests to declare.

## Authors’ contributions

MK cleaned the dataset and contributed to the analysis and manuscript writing. DG contributed to the data analysis, outlining of the manuscript, and technical editing. RG contributed to manuscript writing and, along with OS, provided technical inputs. KL contributed to analysis design, manuscript writing and editing.

## Supporting information


**Additional file 1.** Includes additional information about the Flagship Peer Navigators, including their demographics, sub‐national units where employed, and training/instructions.
