# Supplementary material for: Social network methods for HIV case‐finding among people who inject drugs in Tajikistan
Source: J Int AIDS Soc. 2018 Jul 22;21(Suppl Suppl 5):e25139. doi: 10.1002/jia2.25139 (PMC6055120; doi:10.1002/jia2.25139)
Supplement: Supplementary file 1 — Additional file 1. Includes additional information about the Flagship Peer Navigators, including their demographics, sub‐national units where employed, and training/instructions. [file JIA2-21-e25139-s001.docx]

**Supplemental Text 1:** During the period under analysis 98 Peer Navigators (PNs) were employed. 64% of PNs were males and 36% were females. The age of PNs ranged from 25-45 years old. PNs worked in three main sub-national units: Dushanbe City, Districts of Republican Subordination (Rudaki, Vakhdat, Hissar, Turzunzoda, Shahrinav) and Sughd Oblast (Khudjand, Penjikent, Istaravshan, Isfara and Kanibadam). PNs were assigned to certain regions/neighborhoods in big cities to conduct outreach and HIV testing, while there was not such a division in smaller districts. PNs tended to have existing social ties in the communities where they conducted outreach, but there were additional sites included in the program where PNs needed to establish new connections. PNs were instructed to properly investigate new areas and to collect information about PWID in the area (e.g. injecting sites). Then they created connections and trust with the new group and worked to motivate new clients for HIV testing. PNs were paid a regular salary for their work and were not offered additional performance incentives during the period under analysis, though an incentive scheme was started in October 2017 in an attempt to improve program performance.
